# Missed urethral duplication associated with hypospadias, post-hypospadias repair urethral stenosis, and recurrent urinary tract infections in an adult: a case report

**DOI:** 10.1186/s13256-022-03580-8

**Published:** 2022-09-30

**Authors:** Jalil Hosseini, Saeid Abouei, Alimohammad Mirjalili

**Affiliations:** 1 Men’s Health and Reproductive Health Research Center, Shahid Beheshti University of Medical Sciences, Tehran, Iran; 2 Andrology Research Center, Yazd Reproductive Science Institute, Shahid Sadoughi University of Medical Sciences, Yazd, Iran

**Keywords:** Urethra, Duplication, Abnormalities, Surgical approach

## Abstract

**Background:**

Duplication of urethra is a very rare congenital disorder. Several types of this anomaly have been reported around the world, and are also discussed in this report. However, the mechanism of this anomaly is still unclear.

**Case:**

A 45-year-old Persian man with a complaint of recurrent urinary tract infection was referred to our clinic. He had a history of repairing penoscrotal hypospadias in childhood along with obstructive and irritating symptoms in adolescence. On his last voiding cystourethrogram and retrograde urethrogram, stenosis was observed in the proximal bulbar urethra along with a double urethra in the dorsal region of the main urethra. The double urethra was removed with operation, and he was followed for 1 month after surgery. He had no complaints of recurrence or urinary incontinence.

**Conclusions:**

This report shows the different classification systems, types of double urethra, and approach and management, which mainly involves surgery; however, surgical management should be done according to the anatomical findings of the abnormality.

## Introduction

Urethral duplication is a very rare congenital disorder. Several types of this anomaly have already been described [1]. It occurs predominantly in males; however, a few cases have been reported in women [2]. The mechanism of this disorder is still unclear [3]. Here we report a case of urethral duplication in a 45-year-old man who presented with urethral tract infection (UTI) and urethral stenosis.

## Case presentation

**Fig. 1:**
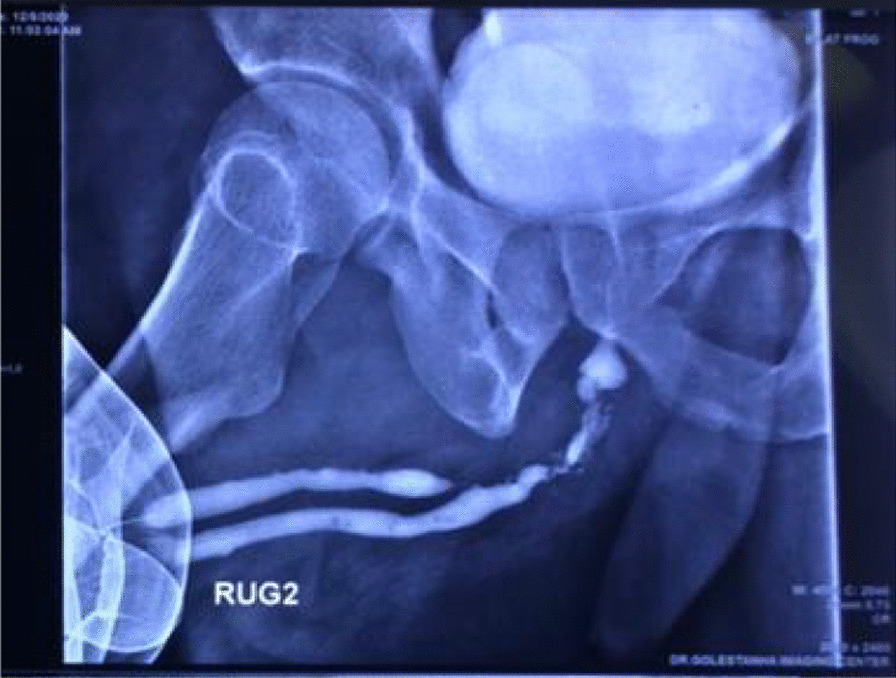
Patient’s RUG before operation, showing stenosis in the proximal region of the bulbar urethra

A 45-year-old Persian man with recurrent urinary tract infection was referred to our clinic. He had a history of penoscrotal hypospadias in childhood, which were repaired, and obstructive and irritating symptoms in adolescence. The patient complained of recurrent urethral tract infection UTI (urine culture was positive for *Escherichia coli*) accompanied with purulent discharge from urethra and fever. He was also referred to other clinics several times over the years and underwent urethra dilatation owing to bulbar tract stenosis. In his last combined voiding cystourethrogram (VCUG) and retrograde urethrogram (RUG), stenosis was observed in the proximal bulbar urethra along with a double urethra in the dorsal region of the main urethra (Fig. 1). On main urethral cystoscopy, the mucosa was relatively pale and the opening of the second urethra was not visible in the penile and bulbar urethra. Stricture and paleness of the mucosa was also seen in the proximal bulbar urethra. On flexible cystoscopy, the opening of the second urethra was visible at 12 o’clock and a distance of 3 cm from the verumontanum in the bulbar urethra. The guidewire was first passed, and it was palpated along the penis at the dorsal level of the patient’s main urethra to a distance of 5 cm from the tip of the penis.

**Fig. 2:**
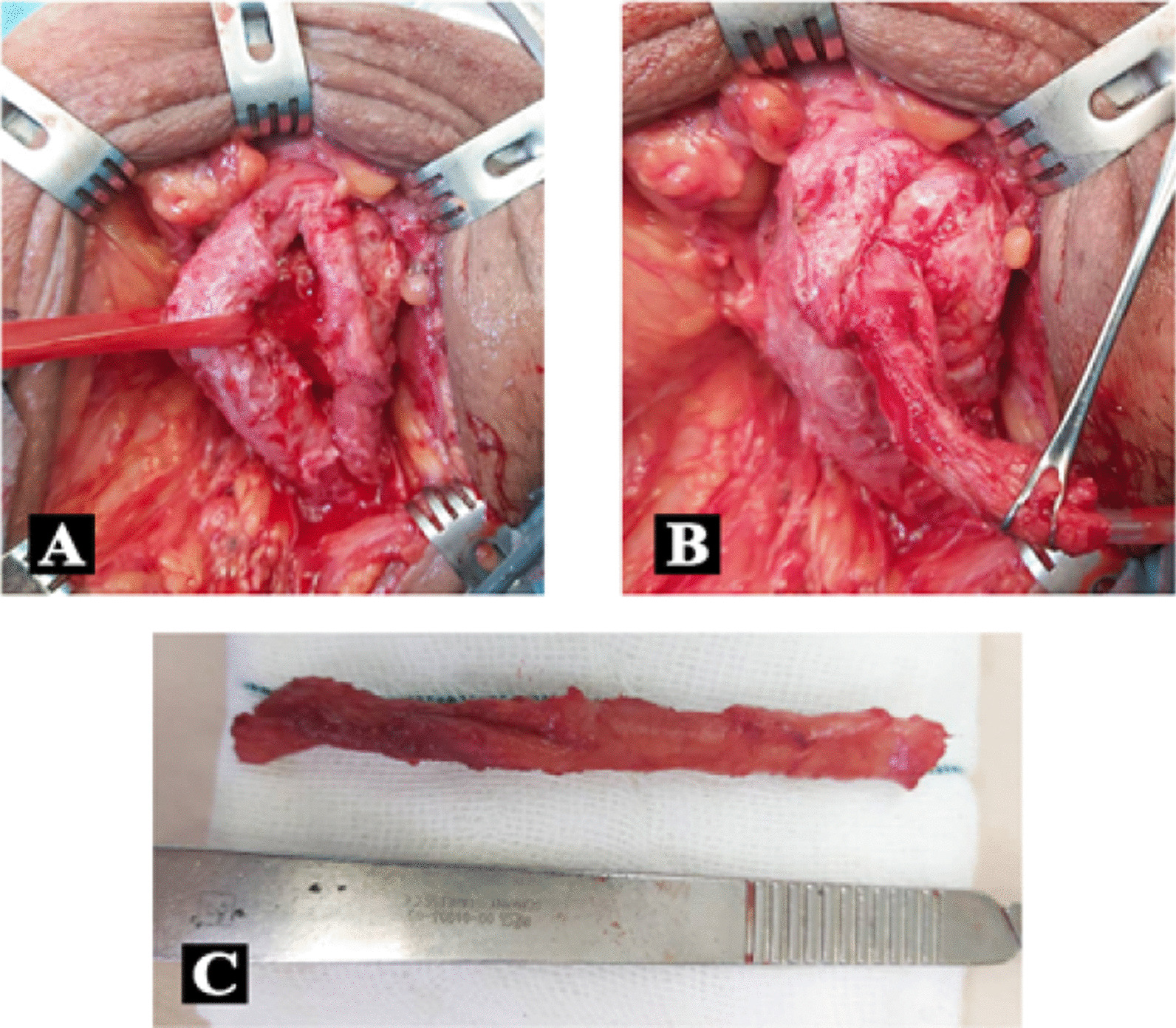
Urethral duplication

The patient was taken to the operation room with a diagnosis of double urethra and underwent general anesthesia in lithotomy position. Then a longitudinal incision was made in the perineal area, and the urethra was dissected from one side. Owing to the stenosis and adhesions caused by previous surgeries, it was released from the dorsal surface and from the corpus cavernosum. The guidewire was then palpated from the dorsal side of the urethra, a plane was taken between the two urethras, and the urethral duplication was dissected from the main urethra. It was removed from the proximal opening of the urethra, and its orifice was repaired with 4–0 vicryl suture. The distal urethra in the dorsal region was completely closed, so it was dissected and the double urethra (secondary) was removed with operation. The patient was followed for 1 month after surgery (Fig. 2). Foley 18 F and 16 F cystostomy catheters were inserted to ensure urinary flow and prevent restenosis. Finally, the skin and fascia were repaired with 4–0 vicryl suture.

Finally, the operation site was bandaged, and the case was taken to the recovery ward with stable vital signs. During hospitalization, urine analysis (UA) and urine culture (UC) tests were performed regularly, which were all normal. The patient was discharged from hospital. He had no complaints of recurrence or urinary incontinence. One month after discharge, RUG showed no evidence of urinary leakage (Fig. 4) and the patient’s UA and UC were reported to be normal.

## Discussion

**Fig. 3:**
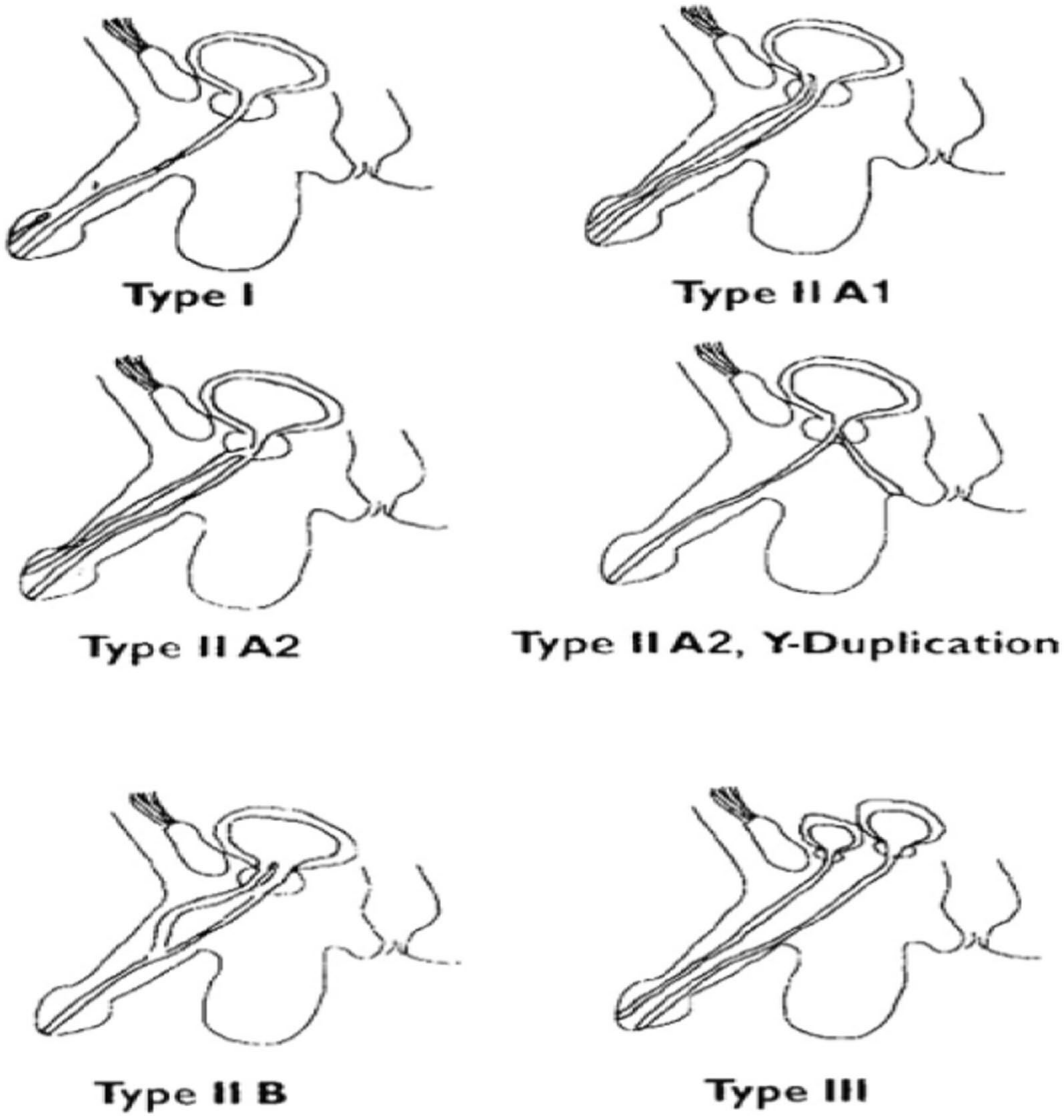
Urethral duplication urinary division

About 10% of newborns have abnormalities of the genitourinary system. One of these rare abnormalities for which a definitive answer has not been found yet is double urethra. In most cases, double urethra occurs in the sagittal plane, and it is divided into dorsal and ventral groups according to the ectopic urethra position. This abnormality is more common in men than women, and unlike other abnormalities, it is rarely associated with other congenital anomalies. Epispadias meatus can develop anywhere in the penis midline [4]. In our case, the main urethra was associated with penoscrotal hypospadias, which was repaired with scrotal skin flaps in childhood. However double urethra was not diagnosed before hypospadias surgery. The patient suffers from urine obstructive and irritative symptoms and alternating UTIs over time and was treated for prostatitis and urethra stricture on several visits to doctors. However, none of the treatments solved the patient’s problem. Owing to recurrent relapse, urethra cystoscopy and dilatation were performed to relieve urethral stricture, but it still continued after the antibiotics were stopped. Finally, the patient was referred to a subspecialty center, where the diagnosis of urethral duplication was confirmed by performing VCUG and RUG. The most common classification for urethral duplication is the Effman classification, in which urethral duplication is divided into three types [5, 6] (Fig. 3):

- **Type I:** incomplete duplication
- I-A. Distal
- I-B. Proximal
- **Type II:** Complete urethral duplication
- II-A1. Two entire urethras
- II-A2. Common urethra at the neck, then separated out as two urethras
- II-A2Y. Here one urethra opens into the rectum
- II-B. Starts as two separate urethras but fuses distally somewhere in its course
- **Type III:** Urethral duplication as a component of partial or complete caudal duplication

Our patient’s urethral duplication was type II-A2, which is less likely to occur than others. Most of the patients with urethral duplication are asymptomatic or visit urology clinics with frequent urination. In some cases, patients find out about the abnormality late in life; in our case, this abnormality was noticed in almost middle age (45 years old). In the case of urethral duplication, imaging techniques should be requested to determine the anatomy, urethra function, and associated abnormalities. Identifying and maintaining a functional urethra is very important in these cases. In many cases, the urethra, which is in a normal position, may be hypoplastic, while the functional urethra is ectopic. The functional urethra has a suitable caliber of urine for complete emptying of the bladder, a good sphincter mechanism, and a normal verumontanum. VCUG shows both ureters in many cases and may show a larger and more continental functional urethra. RUG may be required when the lateral urethra is not visible owing to hypoplasia [7]. Intravenous pyelogram (IVP) or ultrasound can also be used to assess the upper urinary tract.

## Conclusion

**Fig. 4:**
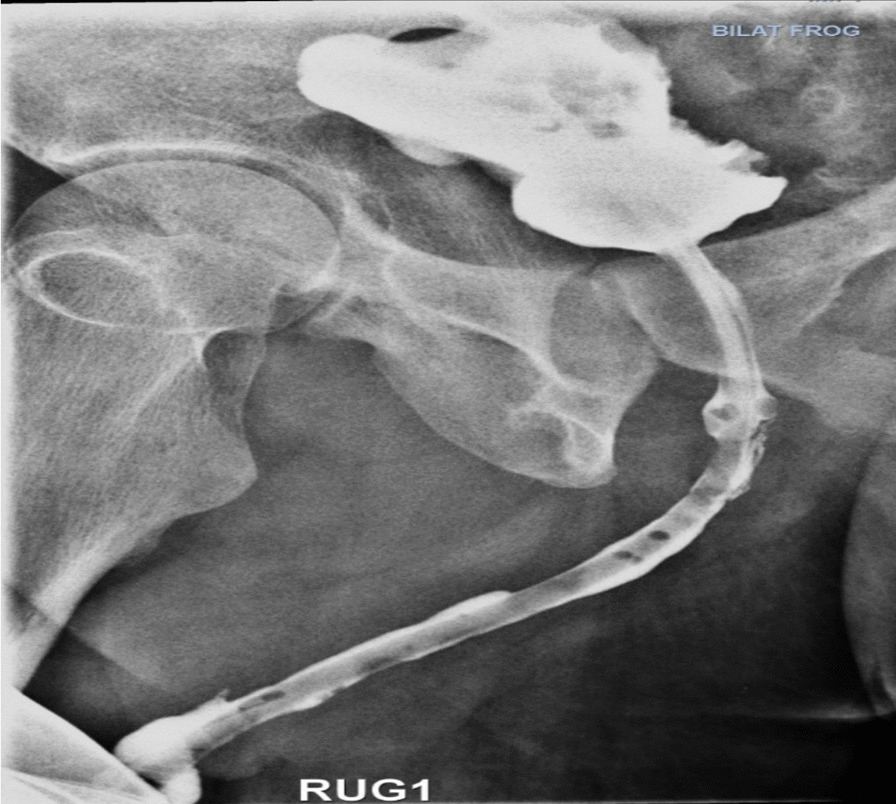
Postoperative RUG

This report shows the different classification systems, types of double urethra, and approach and management, which mainly involves surgery. Surgery can also be used in cases where there are significant cosmetic problems. To prevent external sphincter (patient incontinence) damage, surgery is limited in this type of urethral duplication. A variety of surgical techniques have been described to correct sagittal urethral duplication. To repair type II-A lesions, the patient’s anatomy can be corrected by ventral-to-dorsal ureterostomy or by removal of the urethra with or without urethroplasty (Fig. 4) [8]. As mentioned, complete removal of the urethra is very delicate because it carries the risk of damaging the external sphincter. In cases where the functional urethras are insufficiently caliber or severely hypospadias, one or two stages of urethroplasty may be required for type II-A2 lesions.

## Data Availability

Data regarding any of the subjects in the study have not been previously published unless specified. Data will be made available to the editors of the journal for review or query upon request.
